# Informing the research agenda for optimizing audit and feedback interventions: results of a prioritization exercise

**DOI:** 10.1186/s12874-020-01195-5

**Published:** 2021-01-13

**Authors:** Heather L. Colquhoun, Kelly Carroll, Kevin W. Eva, Jeremy M. Grimshaw, Noah Ivers, Susan Michie, Jamie C. Brehaut

**Affiliations:** 1 Department of Occupational Science and Occupational Therapy, University of Toronto, 160-500 University Ave, Toronto, Ontario M5G 1V7 Canada; 2 Ottawa Hospital Research Institute, Clinical Epidemiology Program, Centre for Practice Changing Research, The Ottawa Hospital, General Campus, 501 Smyth Road, Ottawa, Ontario K1H 8L6 Canada; 3 Centre for Health Education Scholarship, Department of Medicine, University of British Columbia, Vancouver, BC V5Z 4E3 Canada; 4 Department of Medicine, University of Ottawa, 451 Smyth Road, Ottawa, Ontario K1H 8M5 Canada; 5 Department of Family Medicine, Women’s College Hospital, Toronto, ON M5S 1B2 Canada; 6 Department of Clinical, Educational and Health Psychology, University College London, London, WC1E 6BT UK; 7 School of Epidemiology and Public Health, University of Ottawa, 451 Smyth Road, Ottawa, Ontario K1H 8M5 Canada

**Keywords:** Audit and feedback, Implementation science, Knowledge translation, Theory, Research agenda

## Abstract

**Background:**

Audit and feedback (A&F) interventions are one of the most common approaches for implementing evidence-based practices. A key barrier to more effective A&F interventions is the lack of a theory-guided approach to the accumulation of evidence. Recent interviews with theory experts identified 313 theory-informed hypotheses, spread across 30 themes, about how to create more effective A&F interventions. In the current survey, we sought to elicit from stakeholders which hypotheses were most likely to advance the field if studied further.

**Methods:**

From the list of 313, three members of the research team identified 216 that were clear and distinguishable enough for prioritization. A web-based survey was then sent to 211 A&F intervention stakeholders asking them to choose up to 50 ‘priority’ hypotheses following the header “*A&F interventions will be more effective if…”*. Analyses included frequencies of endorsement of the individual hypotheses and themes into which they were grouped.

**Results:**

68 of the 211 invited participants responded to the survey. Seven hypotheses were chosen by > 50% of respondents, including A&F interventions will be more effective… “*if feedback is provided by a trusted source”*; “*if recipients are involved in the design/development of the feedback intervention”*; “*if recommendations related to the feedback are based on good quality evidence”*; “*if the behaviour is under the control of the recipient”*; “*if it addresses barriers and facilitators (drivers) to behaviour change”*; “*if it suggests clear action plans”*; and “*if target/goal/optimal rates are clear and explicit”*. The most endorsed theme was *Recipient Priorities* (four hypotheses were chosen 92 times as a ‘priority’ hypotheses).

**Conclusions:**

This work determined a set of hypotheses thought by respondents to be to be most likely to advance the field through future A&F intervention research. This work can inform a coordinated research agenda that may more efficiently lead to more effective A&F interventions.

**Supplementary Information:**

The online version contains supplementary material available at 10.1186/s12874-020-01195-5.

## Background

Audit and feedback (A&F) interventions involve summarizing data about specific aspects of practice and feeding it back to practitioners to encourage practice change. As one of the most common strategies for aligning healthcare provider behaviour with best evidence [1], A&F interventions are well studied and known to be effective; the most recent Cochrane review of 140 randomized trials showed modest effectiveness (mean of 4% improvement compared to control) across a wide range of applications and settings [1]. However, significant variability in effect sizes (interquartile range 0.5 to 16%) suggests that larger and more consistent effects could likely be achieved with a better understanding of how A&F interventions work [1]. Evidence that A&F intervention effect sizes have not significantly improved since 2003 [2] suggests that this potential is not yet being realized.

We have argued that a key barrier to progress in designing more effective A&F interventions has been the lack of consistent theory to organize and facilitate accumulation of evidence [3, 4]. While many disciplines study feedback (e.g., Social Psychology, Organizational Psychology, Education, Human Factors, Medical Education, Economics, Management), the language and constructs discussed are often not easily applied to a health care setting [5]. Within the health setting, theory appears minimally used, and inconsistently invoked in the actual design of healthcare A&F interventions [3]. In an effort to better understand how theory could be beneficially used in this domain, we previously conducted 28 in-depth interviews with international theory experts from the disciplines listed above, from which we developed 313 testable, theory-informed hypotheses about how to improve A&F interventions [4]. Beyond identifying a small set of issues that seemed uncontroversial and likely to improve A&F interventions immediately [5], we could not rank the relative importance of the 313 hypotheses, nor prioritize which to study further, because of the qualitative and cross-disciplinary nature of the procedure used to collect them [4].

The current analysis sought to begin to prioritize among these hypotheses by asking a group of A&F intervention stakeholders to assess which theory-motivated hypotheses would be most likely to advance the field if prioritized for future research. By identifying the hypotheses thought to be the highest priority by these stakeholders, we hope to accelerate the testing of key questions, create a more coordinated approach to advancing this field, and more efficiently lead to more effective A&F interventions.

## Methods

We conducted a web-based survey that asked stakeholders to prioritize among previously identified theory-motivated hypotheses [4]. Ethics approval was obtained from the Ottawa Health Sciences Network Research Ethics Board. The informed consent process was described in a participant information sheet as being obtained by clicking the survey link. We applied the Checklist for Reporting Results of Internet E-Surveys (CHERRIES) to report this study [6].

### Participants

We defined our sampling frame to be 1) researchers with experience in studying or developing A&F interventions, 2) methodologists from organizations who routinely provide A&F interventions, and 3) knowledge users with specific expertise in A&F interventions. Using participant lists from several international meetings of A&F intervention science and implementation, we were able to generate a list of 211 individuals (75 male, 136 female) coming primarily from Canada (66%), the UK (27%), and other countries (7%).

### Developing the prioritization list

Our previous work identified 313 hypotheses suggested by experts to be testable, theory-informed predictions for how health care A&F interventions could be improved [4]. The list of hypotheses was organized into a set of 30 themes using independent assignment of codes in an iterative process by three coders and confirmed by a fourth member of the team. The resulting hypothesis list was comprehensive, but efforts to translate the list into a survey made it evident that many hypotheses were conceptually similar, had redundant phrasing, or were not entirely clear. As these items would be difficult to prioritize, we undertook a process aimed specifically at eliminating these issues. First, two independent reviewers (HLC;KC) reviewed the full list of hypotheses to group together similar hypotheses and highlight unclear hypotheses. Next, three members of the team (HLC;KC;JCB)  held consensus discussions to confirm unclear and redundant hypotheses and select which hypothesis from any grouping of similar hypotheses was the most clearly worded. In doing so, 98 hypotheses were deleted and one hypothesis was split into two. This process also resulted in a reduction of the number of themes from 30 to 29. The remaining 216 hypotheses were then reviewed again by all three team members for clarity, which led to examples being added to four hypotheses, and a rewording of 15 hypotheses. An example of redundancy was the following two hypotheses in which the first one was retained and the second one eliminated: ‘*Feedback will be more effective when focused on the few, most important behaviours*’; ‘*Feedback will be more effective if the focus is on only one specific behaviour at a time’*. An example of a vague (and therefore eliminated) hypothesis was ‘*Feedback needs to consider alternatives and substitutes beyond the one focal intervention*’. Lastly, the following is a sample hypothesis that was altered to include an example: ‘*Feedback will be more effective if emphasis is on what needs to be achieved (loss framing) as opposed to what was achieved (gain framing)*’ was reworded to include this example: *‘i.e., 20% of your patients did not receive the proper prescription vs. 80% did receive the proper prescription’.* In our previous work, when developing the hypotheses as well as the resulting themes, we used the term ‘feedback’ aiming to refer to the specific data provision episode within the A&F intervention. While the broader term ‘A&F intervention’ could also include other components of a complex intervention, we expect that our use of the term ‘feedback’ in the context of specific interventions was generally interpreted as a placeholder for the term ‘A&F intervention’. In this manuscript, we use the term ‘A&F intervention’ to refer to these interventions but have retained the term ‘feedback’ when describing hypotheses, the themes and the study materials in the Additional files 1 and 2.

### Survey design

In designing the prioritization exercise, our goal was to identify a list of hypotheses that members of the A&F intervention research community believe should be prioritized for further exploration. Because it would have likely had an adverse affect on response rate to ask participants to rank order all 216 hypotheses, we asked them to choose a list of up to 50 ‘priority’ hypotheses. We chose the number 50 to achieve a reasonable trade-off between restrictiveness (‘why can’t I choose this important additional hypothesis?’) and specificity (i.e., requiring respondents to be more selective than a simple yes/no endorsement).

The online survey was developed for this study, and created by and housed at the Ottawa Hospital Research Institute. See Additional file 2 for a copy of the survey. It consisted of four tabs that respondents could select sequentially: Instructions, Demographics, Prioritization Exercise, and Summary. Instructions asked respondents to consider 1) the quality of the idea behind the hypothesis (as best as they could interpret it), and 2) its likelihood of advancing the field. If they thought a hypothesis was interesting but poorly worded, they were instructed that they could still select the hypothesis but should provide comments about wording and why they selected it despite the problem identified. Respondents were also instructed not to select hypotheses that they felt were unclear, uninteresting, or already well understood.

Demographics collected on respondents included Country where the respondent does most of their work (via text box), Work Role (check all that apply: Researcher, Policy Maker, Health System Administrator, Healthcare Delivery, Other), and Career Level (via drop-down menu: Early (< 5 years); mid (5–15 years) and senior (> 15 years)). As the survey group was known to our team (i.e., names gathered from invited meetings), we extracted Sex and Country variables for the entire sample frame based on existing meeting information; identifying information was eliminated from the dataset prior to analysis.

The prioritization exercise included a series of web pages (~ 10 hypotheses per page) that highlighted the theme from which each hypothesis was derived, hypothesis number, the hypothesis itself, a checkbox for selecting that hypothesis as one of the top 50, and an optional comment box next to each hypothesis. We included a function on the prioritization tab to reveal a running tally of how many hypotheses had been selected (i.e., ‘35 of 50 selected’). If more than 50 hypotheses were selected, a pop-up message appeared with a reminder to limit selection to 50. If more than 60 were selected, a warning message appeared that the maximum number had been selected. That is, while participants were told to choose 50 hypotheses, they could select any number up to 60. Theme presentations were randomly sorted for each participant to reduce order effects that might have arisen due to respondent fatigue, but hypotheses within each theme remained in a consistent order to facilitate clarity of the theme.

The summary tab listed all chosen hypotheses, relevant themes, and the total number of hypotheses selected. Respondents were able to review their selections and make changes as needed. If they were happy with their selections, they were instructed to click the “all done” button at the bottom of the summary page.

The task was piloted in two phases. An initial beta test (in Microsoft Excel) was carried out among team members (HLC;KWE,NI;SM;JCB)  to ensure the instructions were clear, that the task was easy to complete, and to get a sense of the time commitment involved. After final web programming, the survey was again pilot tested among the team (HLC;KC;JCB)  to ensure ease of use, understandability, and functionality.

### Survey administration

Participants were sent an invitation email from the study PI on January 9, 2018 that included a short description of our work leading up to this prioritization survey, information on the task at hand, a unique participant ID with password, and the web survey link. The email also listed the names of our study team and included a published paper about our work [5] along with the REB approved participant information sheet as attachments. The participant information sheet included all regulatory requirements, such as the study purpose, funding information, and how personal information would be protected. Participants were told that the survey was voluntary and would likely take no more than 60 min to complete. Non-responders were sent three follow-up emails at approximately 2-week intervals (i.e., January 22, 2018, February 13, 2018 and March 9, 2018). Duplicate entries were avoided by assigning unique participant IDs with a password, managing password resets through a research coordinator, and having the participant re-enter the survey at the point where they last exited if they logged in multiple times.

### Analysis

The data were downloaded from the locally hosted secure server into Microsoft Excel for analysis. Frequencies were calculated for all demographic variables and for Sex and Country, with chi-squared analyses calculated to evaluate differences between responder and non-responder groups.

We calculated the total number of times individual hypotheses, and hypotheses in each theme, were endorsed in respondents’ top 50. As the number of hypotheses in each theme varied, our theme endorsement was calculated as a proportion of possible endorsements (i.e., number of endorsements of hypotheses in a theme over the number of hypotheses in the theme multiplied by the number of participants).

### Post hoc sub-group analysis

As a test of the robustness of the rankings and to determine if researchers had different priorities from non-researchers, we conducted a post hoc sub-group analysis of the correlation between the two sets of rankings by calculating the percentage of time each hypothesis was endorsed by members of each subgroup.

## Results

Sixty-eight respondents began the survey, for a response rate of 32.2% (68/211). Seven of the 68 respondents, however, did not complete any of the prioritization exercise, leaving 61 complete surveys and a participation rate of 28.9%. Table 1 describes their characteristics. Chi-squared analyses indicated that a higher proportion of men (29/46; 63.0%) responded than did women (32/104; 30.8%), χ^2^_(1)_ = 5.4, *p* = .02, but no significant differences were found related to respondent country, χ^2^_(2)_ = .28, *p* = .87. The majority of respondents were from Canada (*n* = 39; 64%) with the UK being the next most common country (*n* = 17; 28%). The majority had a self-described role as a researcher (*n* = 34; 56%) and about half (48%) indicated they were at a senior career stage. While most (56/61) of the respondents chose 50 hypotheses, there were five who chose a different number (19, 24, 44, 46, and 51 hypotheses).

**Table 1:** Responder characteristics with chi-squared for differences between responder and non-responder for two characteristics, *n* = 61 responders, *n* = 150 non-responders

Characteristic		Responders Number (%)	Non-Responders Number (%)	chi-square statistic (***p*** value)
Country	Canada	39 (64%)	101 (67%)	
	United Kingdom	17 (28%)	39 (26%)	
	Other	5 (8%)	10 (6%)	χ^2^_(2)_ = .28, p = .87
Gender	Male	29 (48%)	46 (31%)	
	Female	32 (52%)	104 (69%)	χ^2^_(1)_ = 5.4, p = .02
Self-Described Role	Researcher	34 (56%)		
	Policy Maker	2 (3%)		
	Health System Administrator	8 (13%)		
	Healthcare Delivery	3 (5%)		
	Other (e.g., medical education,	12 (20%)		
	Not completed	2 (3%)		
Career Stage	Mid-career	23 (38%)		
	Early/New	6 (10%)		
	Senior	29 (48%)		
	Not completed	3 (5%)		

Additional file 1 provides a list of all hypotheses, rank ordered by the number of participants who chose each as one of their top 50. As an arbitrary, but more manageable subset, Table 2 provides a summary of the seven hypotheses endorsed by a majority (> 50%) of our respondents. These seven hypotheses (each of which was preceded by the header *“Feedback interventions will be more effective….”)* are as follows: “*if feedback is provided by a trusted source”; “if recipients are involved in the design/development of the feedback intervention”; “when recommendations related to the feedback are based on good quality evidence”; “if the behaviour is under the control of the recipient”; “if it addresses barriers and facilitators (drivers) to behaviour change”; “if it suggests clear action plans”;* and *“when target/goal/optimal rates are clear and explicit”.* Examples of poorly endorsed hypotheses included: “*if for low self-esteem individuals, negative feedback does not follow positive feedback”; “when guidance specifically addresses the sign of the feedback for that individual”; “when not limited to correct/incorrect evaluations”;* and *“if it includes an unconditional incentive”.* The latter two hypotheses were never chosen.

**Table 2:** Summary of ‘Top Hypotheses’ (i.e., those voted as one of the top ‘50’ by > 50% of the sample)

Hypotheses (theme)	Number of participants who chose this hypotheses (%)
**A&F interventions will be more effective…**	
1. …if the feedback is provided by a trusted source (Trustworthiness/Credibility)	45 (74%)
2. …if recipients are involved in the design/development of the feedback intervention (Decision Processes or Conceptual Model)	37 (61%)
3. …when recommendations related to the feedback are based on good quality evidence (Trustworthiness/Credibility)	37 (61%)
4. …if the behaviour is under the control of the recipient (Self -Efficacy/Control)	35 (57%)
5. …if it addresses barriers and facilitators (drivers) to behaviour change (Remove Barriers)	33 (54%)
6. …if it suggests clear action plans (Enable Action Plans/Coping Strategies)	32 (52%)
7. …when target/goal/optimal rates are clear and explicit (Goal Setting)	31 (51%)

Table 3 provides a summary of the most consistently endorsed themes, including variation in number of hypotheses per theme. Few themes were universally endorsed: the proportion of possible endorsements ranged from a maximum of 38% (*Recipient Priorities*, e.g., ‘*A&F interventions will be more effective when recipients believe that the target behaviour needs to change*’), to a minimum of 9% (*Recipient Characteristics*, e.g., ‘*A&F interventions will be more effective if it incorporates an understanding of the communication style of the recipient’*).

**Table 3:** Summary of hypotheses chosen by theme, ordered by proportion of possible endorsements

Theme Name (number of hypotheses in theme)	Potential endorsement (number of hypotheses in the theme × 61 participants)	Actual endorsement (number of times hypotheses in theme were chosen as top 50)	Proportion (%) of possible endorsements
1. Recipient Priorities (4)	244	92	38%
2. Decision Processes or Conceptual Model (3)	183	65	36%
3. Justify Need for Behaviour Change (3)	183	61	33%
4. Environment (3)	183	59	32%
5. Self-Efficacy/Control (5)	305	97	32%
6. Trustworthiness/Credibility (11)	671	205	31%
7. In-Person Feedback (1)	61	18	30%
8. Attack on Self-Identity (6)	366	94	26%
9. Social Engagement (10)	610	157	26%
10. Attract/Maintain Attention (5)	305	76	25%
11. Goal Setting (13)	793	197	25%
12. Responding to Feedback Providers (2)	122	30	25%
13. Cognitive Load (21)	1281	302	24%
14. Comparisons (18)	1098	251	23%
15. Feedback Specificity (10)	610	134	22%
16. Single Hypotheses (10)	610	134	22%
17. Cognitive Influences (4)	244	52	21%
18. Motivation/Intention Issues (11)	671	136	20%
19. Guide Reflection (6)	366	74	20%
20. Memory (6)	366	72	20%
21. Feedback Timing (14)	854	165	19%
22. Enable Action Plans/Coping Strategies (6)	366	70	19%
23. User-Guided Experience (4)	244	46	19%
24. About Aspects of Behaviour (7)	427	80	19%
25. Nature of Data (5)	305	54	18%
26. Opportunity Costs (3)	183	31	17%
27. Remove Barriers (7)	427	65	15%
28. Knowledge/Learning (9)	549	82	15%
29. Recipient Characteristics (9)	549	47	9%

### Post hoc sub-group analysis

The researcher and non-researcher rankings were found to be highly positively correlated, r(214) = .710, *p* = .000, suggesting considerable consistency in the rankings between the two groups.

## Discussion

A survey administered to A&F intervention stakeholders has resulted in 216 hypotheses ordered by number of endorsements as to the quality of the idea and its plausibility of advancing the field. The list could facilitate the development of a coherent, theory-guided research agenda for optimizing A&F interventions for implementation. Seven hypotheses were endorsed by at least 50% of our participants, suggesting they might be prioritised for future testing. The three themes most endorsed through selection of an associated hypothesis were *Recipient Priorities* (i.e., hypotheses relevant to how A&F interventions can be aligned to issues that are important to recipients); *Decision Processes or Conceptual Model* (i.e., hypotheses relevant to better understanding how decisions based on A&F interventions are made); and, *Justify Need For Behaviour Change* (i.e., hypotheses relevant to effectively integrating a rationale for behaviour change into A&F interventions). Hypotheses within these themes provide examples of how to further explore these important issues.

Preparing our list of the original 313 hypotheses [4] for prioritization resulted in an unexpectedly large reduction in rankable hypotheses (i.e., 313 hypotheses reduced to 216), and despite the effort engaged to do so, the variation in the clarity and complexity of the remaining items endured. We see this as stemming from the challenging and imperfect process of extracting theoretical ideas from qualitative discussions [4, 5] (i.e., the difficulty of converging such into simple statements of testable hypotheses). An expert consensus process would likely be needed to create a smaller, clearer list of ideas that bridge jargon differences across disciplines while still summarising the wealth of theoretical information available around providing effective A&F interventions.

The effort to identify hypotheses that could be used in a prioritization process, however, offers useful guidance regarding future directions that might help to coordinate A&F intervention research. For example, the existing Cochrane review, which was completed a full decade ago, outlines only five A&F intervention characteristics associated with effectiveness (i.e., source is a supervisor or colleague; is delivered more than once; is verbal and written; aims to decrease undesirable behaviour as opposed to increase desirable behaviour; and includes explicit targets and action plans). An updated Cochrane Review (currently underway) on the effectiveness of A&F interventions would be well-informed by evaluating the other conditions our participants have highlighted as needing to be studied to determine how to engage in effective A&F interventions [5]. Similarly, efforts like the A&F Metalab, a network facilitating collaboration between healthcare organizations that deliver A&F interventions to complete large-scale trials [7], might have their work made more efficient by prioritizing hypotheses thought to yield most promise for improving A&F interventions.

Systematic priority setting in implementation science is relatively new, but we propose that such efforts are necessary to improve the impact, resource allocation and progression of the science. In addition to our methods outlined here, other examples of priority setting in the field include surveys of implementation science trainees to establish research and practice priorities [8], the use of Nominal Group Technique to prioritize gaps in evidence and practice [9], and evidence collection to support the impact of incorporating patients in priority setting in health delivery [10]. Future efforts to expand and evaluate priority setting activities throughout the field would be beneficial.

### Limitations

A number of limitations of this work warrant consideration. First, it must be acknowledged that rankings of themes with relatively few hypotheses will be less stable than those with more hypotheses. Second, we cannot rule out that, despite our instructions, respondents may have considered factors other than ‘how the hypothesis would advance the field’ when providing their endorsements. Informal feedback from respondents suggests that some endorsements about advancing the field may have actually included thinking both about the scientific value of a hypothesis as well as whether they ‘agreed’ with the hypothesis, an explanation given more weight given that hypotheses related to ‘trusted sources’ were often chosen despite this being a clear finding in the existing literature [1]. In addition, despite our efforts to ensure clarity of items, it is possible that clarity influenced the rate of endorsement (e.g., if ‘…*recipients are involved in the design/development of the feedback intervention*’ is more clear than ‘…*when not limited to correct/incorrect evaluations*’, it may have influenced the rate at which both were selected). Thus, we propose that while these rankings can inform research priorities, they should be treated as guidance to be considered alongside existing evidence rather than the sole basis for determining what hypotheses deserve most empirical attention.

In addition, while this work does represent the largest sample of participants engaged in A&F intervention priority-setting to date, our participation rate was 29%, suggesting that it might not be representative of the full sample frame. We may have increased our participation rate had we engaged in additional incentives to participate, however the greater likelihood was that the challenging and time-consuming nature of the survey limited participation. Furthermore, the sample frame itself excluded relevant stakeholders from other countries given that it was limited to participants in a series of A&F intervention related meetings. Finally, it is worth noting that our sample was relatively researcher- and male-focused, potentially leading to biases in our prioritization exercise even though we know of no reason that these subgroups should harbour different opinions from non-researchers or females (and, indeed, of those who participated, researcher priorities were associated very strongly with non-researcher priorities).

## Conclusion

The goal of this study was to ask A&F intervention stakeholders to prioritize among previously identified theory-motivated hypotheses to assess which would be most likely to advance the field through further empirical testing.. This work can inform a more coordinated approach to advancing this field and should move us towards interventions that are informed by relevant theory, and may more efficiently lead to more effective A&F interventions.

## Supplementary Information

**Additional file 1. **All 216 hypotheses by rank order with theme (*n* = 61 participants).

**Additional file 2.** Audit and Feedback Hypotheses Prioritization Exercise.

## Data Availability

The datasets generated during and/or analysed during the current study are available from the corresponding author on reasonable request. The dataset supporting the conclusions of this article is included within the article (and its additional file).

## Abbreviation

A&F: Audit and Feedback
