# Supplementary material for: Informing the research agenda for optimizing audit and feedback interventions: results of a prioritization exercise
Source: BMC Med Res Methodol. 2021 Jan 13;21:20. doi: 10.1186/s12874-020-01195-5 (PMC7805176; doi:10.1186/s12874-020-01195-5)
Supplement: Supplementary file 1 — Additional file 1. All 216 hypotheses by rank order with theme (n = 61 participants). [file 12874_2020_1195_MOESM1_ESM.docx]

**Additional file 1**

All 216 hypotheses by rank order with theme (n=61 participants).

| **Hypotheses** | **Theme** | **Number of participants who chose this hypothesis as one of their top 50 (%)** |
| --- | --- | --- |
| **A&F interventions will be more effective…** |  |  |
| 1. …if the feedback is provided by a trusted source | Trustworthiness/Credibility | 45 (74%) |
| 1. …if recipients are involved in the design/development of the feedback intervention | Decision Processes or Conceptual Model | 37 (61%) |
| 1. …when recommendations related to the feedback are based on good quality evidence | Trustworthiness/Credibility | 37 (61%) |
| 1. …if the behaviour is under the control of the recipient | Self -Efficacy/Control | 35 (57%) |
| 1. …if it addresses barriers and facilitators (drivers) to behaviour change | Remove Barriers | 33 (54%) |
| 1. …if it suggests clear action plans | Enable Action Plans/Coping Strategies | 32 (52%) |
| 1. …when target/goal/optimal rates are clear and explicit | Goal Setting | 31 (51%) |
| 1. …when they involve formative (identifying areas to help improve) rather than summative (performance only) assessment | Attack on Self Identity | 29 (48%) |
| 1. …if the environment encourages the desired behaviour as the default | Environment | 29 (48%) |
| 1. …if it is relevant to issues that are a priority for recipients | Recipient Priorities | 28 (46%) |
| 1. …if they encourage co-construction of goals among colleagues | Social Engagement | 28 (46%) |
| 1. …if the recipient agrees that the benchmark is relevant to them | Comparisons | 27 (44%) |
| 1. …if it is structured according to the most relevant data unit (e.g. individual, practice) | Feedback Specificity | 27 (44%) |
| 1. ..when a comparator is provided | Comparisons | 26 (43%) |
| 1. …if the comparator is specific to the recipient's own context/practice | Comparisons | 26 (43%) |
| 1. …if it clearly identifies a behaviour that should be improved | Goal Setting | 26 (43%) |
| 1. …when recipients believe that the target behaviour needs to change | Recipient Priorities | 26 (43%) |
| 1. …if targeted at a small number of the highest priority issues. | Recipient Priorities | 26 (43%) |
| 1. …if they encourage engagement with the data | Attract/Maintain Attention | 25 (41%) |
| 1. …if the goals are believed to be reasonable and attainable | Goal Setting | 25 (41%) |
| 1. …if individual level provider data is provided | Feedback Specificity | 25 (41%) |
| 1. …if they address all relevant members of the practice team, not a single provider | Social Engagement | 24 (35%) |
| 1. …when designed to reduce cognitive load demands (e.g. include more white space, eliminate decimals, clear legend; left to right reading flow) | Cognitive Load | 24 (35%) |
| 1. …if it makes reference to performance successes in addition to providing clear direction on how to improve | Motivation/Intention Issues | 24 (35%) |
| 1. …if it is provided to the intended target for behavior change | Single Hypotheses | 23 (38%) |
| 1. …if the information explaining the audit and feedback is clear and unambiguous | Cognitive Load | 23 (38%) |
| 1. …if educational messages are clearly presented | Cognitive Load | 23 (38%) |
| 1. …if it provides clear direction on the behaviour requiring change | Cognitive Load | 23 (38%) |
| 1. …if the harms associated with incorrect behaviour in question are clearly indicated | Justify Need for Behaviour Change | 23 (38%) |
| 1. …if the reminder messages are presented in real time/point of care | Memory | 23 (38%) |
| 1. …if it can elicit a sense of achievement when a target is reached (achievement motivation) | Motivation/Intention Issues | 23 (38%) |
| 1. …if the benchmark comparison is accepted as a reasonable standard | Comparisons | 22 (36%) |
| 1. …when presented soon after the audited actions are taken | Feedback Timing | 22 (36%) |
| 1. …when justified by improvements in patient care rather than cost savings | Justify Need for Behaviour Change | 22 (36%) |
| 1. …if the recipients have the capabilities to respond to the feedback | Self-Efficacy/Control | 21 (34%) |
| 1. …if they involve social group interaction within a safe/trusted environment | Social Engagement | 21 (34%) |
| 1. …if it is perceived to be without conflict of interest | Trustworthiness/Credibility | 21 (34%) |
| 1. …if (over time) it is accompanied with positive reinforcement to those who have improved their performance | Motivation/Intention Issues | 21 (34%) |
| 1. Feedback interventions involving starting new behaviours will be more effective if they involve reminders/prompts | About Aspects of Behaviour | 21 (34%) |
| 1. …if it contains multi-modal presentation (both text and graphs | Single Hypotheses | 20 (33%) |
| 1. …if a priori work is conducted to ensure acceptability of the benchmark and feedback | Trustworthiness/Credibility | 20 (33%) |
| 1. …if a clear and explicit benchmark is provided | Comparisons | 20 (33%) |
| 1. …when it is presented continuously/as part of regular care | Feedback Timing | 20 (33%) |
| 1. …when placed in the context of real-time comparison with peers | Feedback Timing | 20 (33%) |
| 1. …when it involves goals set/agreed to by the participant | Goal Setting | 20 (33%) |
| 1. …when recipients internalize and act on the feedback, rather than only responding to a system prompt | Guide Reflection | 20 (33%) |
| 1. …if they allow the recipient to respond to the feedback providers | Responding to Feedback Providers | 20 (33%) |
| 1. …if emphasis is on what needs to be achieved (loss framing) as opposed to what was achieved (gain framing) (i.e., 20 % of your patients did not receive the proper prescription vs. 80% did receive the proper prescription | Cognitive Influences | 19 (31%) |
| 1. ...if it focuses on patient outcome measures rather than process measures | Single Hypotheses | 19 (31%) |
| 1. …when it provides information on the appropriateness of individual decisions, not just frequency of behaviours | Feedback Specificity | 19 (31%) |
| 1. …when they incorporate ways to track subsequent actions. | Feedback Timing | 19 (31%) |
| 1. ...if the graphical representations are clearly and consistently labelled. | Cognitive Load | 19 (31%) |
| 1. ...if it involves comparisons to the self. | Comparisons | 19 (31%) |
| 1. ...if designed with a clear understanding of the decision-making process underlying the behaviour to be changed. | Decision Processes or Conceptual Model | 18 (30%) |
| 1. ...if it includes both local and overall norms. | Comparisons | 18 (30%) |
| 1. ...if trend data are sufficiently stable to facilitate interpretation. | Nature of Data | 18 (30%) |
| 1. …when it is in person (can be presented in a manner that is responsive to the situation). | In-Person Feedback | 18 (30%) |
| 1. ...if it creates opportunities to learn. | Knowledge/Learning | 18 (30%) |
| 1. ...if it is expected that recipient behaviour change will result in improvements. | Self-Efficacy/Control | 18 (30%) |
| 1. …if they involve engaging recipients in social discussion about the feedback. | Social Engagement | 18 (30%) |
| 1. …when measures are used to prevent a defensive response (e.g. providing other "reassuring “messages as well, guiding self-reflection, etc.). | Attack on Self-Identity | 18 (30%) |
| 1. …if they target communally determined behaviour change strategies (i.e. the group works together towards tipping points). | Social Engagement | 17 (28%) |
| 1. …if individual level data is worded as a recommendation (e.g., in most cases, doing x is the best course of action) and aggregate level data is prescriptive (e.g., the guidelines states to do x). | Feedback Specificity | 17 (28%) |
| 1. …if resulting patient outcomes over time support behaviour change. | Feedback Timing | 17 (28%) |
| 1. ...when it supports learner-determined rather than externally imposed goals. | Goal Setting | 17 (28%) |
| 1. ...if it involves a personal reflection component. | Guide Reflection | 17 (28%) |
| 1. …if they include memorable/salient messages. | Memory | 17 (28%) |
| 1. ...if it is about a behaviour that does not rely on others. | About Aspects of Behaviour | 17 (28%) |
| 1. ..if disagreement with recommendations are explicitly acknowledged and addressed. | Social Engagement | 17 (28%) |
| 1. ..if it includes multi-layered feedback which begins with high-level feedback and then drills down to the details. | User-Guided Experience | 17 (28%) |
| 1. ...when it does not imply fault. | Attack on Self-Identity | 17 (28%) |
| 1. ...if any social comparisons are perceived as relevant and attainable. | Comparisons | 17 (28%) |
| 1. ...if incorporated into familiar processes of care. | Environment | 17 (28%) |
| 1. …if the goal is made public. | Single Hypotheses | 16 (26%) |
| 1. ...if accompanied by information about the importance of the behavior change. | Justify Need for Behaviour Change | 16 (26%) |
| 1. …if a record of success is established with "early win" goals prior to moving onto more challenging goals. | Trustworthiness/Credibility | 16 (26%) |
| 1. ...when presented in a clear and aesthetically pleasing way. | Attract/Maintain Attention | 16 (26%) |
| 1. ...if key messages are visually distinguished from supporting material. | Cognitive Load | 16 (26%) |
| 1. …when different modes of information (e.g. graphics, text) are complementary, not redundant. | Cognitive Load | 16 (26%) |
| 1. …when text is simplified and minimized. | Cognitive Load | 15 (25%) |
| 1. ...if patient-specific information is provided. | Feedback Specificity | 15 (25%) |
| 1. …if individual members are personally committed to the group goal. | Goal Setting | 15 (25%) |
| 1. ...if it encourages reflection on the original pattern of behaviour. | Guide Reflection | 15 (25%) |
| 1. …if they also incorporate reminders. | Memory | 15 (25%) |
| 1. ...when accompanied by incentive. | Motivation/Intention Issues | 15 (25%) |
| 1. ...if graphical representation displays the variability of data in order to indicate the error or uncertainty (i.e., confidence intervals). | Nature of Data | 15 (25%) |
| 1. …if the recipients of the feedback identify with the messenger of the feedback. | Trustworthiness/Credibility | 15 (25%) |
| 1. …when origin of benchmarks is made clear. | Trustworthiness/Credibility | 15 (25%) |
| 1. ...if more detailed information is available on demand. | User-Guided Experience | 15 (25%) |
| 1. ..if the recipient reads / processes it. | Attract/Maintain Attention | 15 (25%) |
| 1. …if it provides a visually clear target rate. | Cognitive Load | 15 (25%) |
| 1. ...if a response or action is required. | Enable Action Plans/Coping Strategies | 15 (25%) |
| 1. ...when the important comparisons are in proximity to one another. | Cognitive Load | 14 (23%) |
| 1. ...if it is non-punitive. | Attack on Self-Identity | 14 (23%) |
| 1. ...if it is accompanied with a goal. | Goal Setting | 14 (23%) |
| 1. …when specific to patients most likely to benefit from the change in provider behaviour. | Feedback Specificity | 14 (23%) |
| 1. …if they make clear where the recipient is an outlier. | Motivation/Intention Issues | 14 (23%) |
| 1. ...when there are few costs to change behaviour. | Opportunity Costs | 14 (23%) |
| 1. …if they imply some kind of extended commitment (e.g. agreeing to a future communication, follow-up). | Single Hypotheses | 14 (23%) |
| 1. …if it includes stratification by common "alibi" variables (i.e., demonstrating that "my patients are not sicker"). | Single Hypotheses | 14 (23%) |
| 1. …when they incorporate facilitated social discussions about the feedback | Social Engagement | 14 (23%) |
| 1. ...if the interpretation to be drawn from the comparison to benchmark is made clear and explicit. | Cognitive Load | 13 (21%) |
| 1. ...if they include motivational messages that are tailored to the individual provider. | Motivation/Intention Issues | 13 (21%) |
| 1. ...when the practice gap is at least partly caused by a lack of knowledge. | Knowledge/Learning | 13 (21%) |
| 1. …if they incorporate an understanding of the communication style of the recipient. | Recipient Characteristics | 13 (21%) |
| 1. ..if data come from sources similar to the recipient's clinical practice. | Trustworthiness/Credibility | 13 (21%) |
| 1. ...if the focus is on only one specific behaviour at a time. | Cognitive Load | 13 (21%) |
| 1. ...when the reader is oriented to how to read the feedback. | Cognitive Load | 13 (21%) |
| 1. ...if the degree of difference between comparators is clear and made relevant. | Comparisons | 13 (21%) |
| 1. …when multiple individual physician practice data is presented along with the recipients' data. | Comparisons | 13 (21%) |
| 1. ...if it incorporates the typical clinical encounter decisions in the specific context. | Environment | 13 (21%) |
| 1. …if they encourage people to use implementation intention strategies. | Enable Action Plans/Coping Strategies | 12 (20%) |
| 1. …it facilitates respect, feelings of control over the learning agenda. | Self-Efficacy/Control | 12 (20%) |
| 1. …if they provide information sought by the recipient. | Recipient Priorities | 12 (20%) |
| 1. ...if targeted at those who are underperforming. | Recipient Characteristics | 12 (20%) |
| 1. ...if reflection occurs soon after feedback. | Guide Reflection | 12 (20%) |
| 1. ...if each episode of feedback includes multiple time points. | Feedback Timing | 12 (20%) |
| 1. ...when it addresses a behaviour that is relevant to the current patient. | About Aspects of Behaviour | 12 (20%) |
| 1. ...when it can be customized by the recipient. | User-Guided Experience | 12 (20%) |
| 1. ...when it is sufficiently salient and receives sufficient attention. | Attract/Maintain Attention | 12 (20%) |
| 1. …if noun descriptors rather than verbs are used in messaging (e.g., don't be an over prescriber vs please prescribe less | Cognitive Influences | 12 (20%) |
| 1. ...if text accompanying graphical components only describes information clearly related to the graphical content. | Cognitive Load | 12 (20%) |
| 1. ...if only the most critical information is presented initially. | Cognitive Load | 12 (20%) |
| 1. Feedback interventions involving multiple quality indicators will be more effective if the sign is consistent (i.e. higher numbers are better). | Cognitive Load | 11 (18%) |
| 1. …when graphical representations of sub-par performance are displayed below, and good performance displayed above, a visual frame of reference | Cognitive Influences | 11 (18%) |
| 1. ...if it elicits a clear affective response. | Attack on Self-Identity | 11 (18%) |
| 1. ..when recipients believe the change is THEIR idea. | Self-Efficacy/Control | 11 (18%) |
| 1. …when it is available when the recipient is receptive to it (pull), rather than directed to them at a time not of their choosing (push). | Feedback Timing | 11 (18%) |
| 1. ...if it is corrective (what was wrong, how to improve it). | Knowledge/Learning | 11 (18%) |
| 1. …if the frequency of the feedback is determined by the frequency of the target behaviour | Feedback Timing | 11 (18%) |
| 1. …if they include active learning strategies (e.g. simulations, games with feedback). | Knowledge/Learning | 11 (18%) |
| 1. …if they target (triage) individuals who have motivation (intention) to change. | Motivation/Intention Issues | 11 (18%) |
| 1. Feedback about behaviour will be more effective for behaviors that are easy compared to those that are harder to do. | About Aspects of Behaviour | 11 (18%) |
| 1. Feedback interventions involving stopping behaviours will be more effective if they involve persuasive components. | About Aspects of Behaviour | 11 (18%) |
| 1. ...if graphical elements are without unnecessary depth elements. | Cognitive Load | 11 (18%) |
| 1. ..when presenting absolute numbers as opposed to percentages. | Cognitive Load | 11 (18%) |
| 1. …if targets are made aware of the involvement of other stakeholder groups in the development process. | Decision Processes or Conceptual Model | 10 (16%) |
| 1. …if they allow an opportunity to indicate why a recommended action wasn't taken. | Responding to Feedback Providers | 10 (16%) |
| 1. ..if it incorporates messages specifically about barriers to the target behaviour. | Remove Barriers | 10 (16%) |
| 1. ...if opportunity costs of engaging with the feedback are taken into account. | Opportunity Costs | 10 (16%) |
| 1. ...if there is an immediate cue to action, during the patient encounter. | Feedback Timing | 10 (16%) |
| 1. …if the goal is above current performance. | Goal Setting | 10 (16%) |
| 1. ..when the comparator depicts the goal rather than a peer comparison. | Goal Setting | 10 (16%) |
| 1. …if they involve demonstrations of the behaviour. | Single Hypotheses | 10 (16%) |
| 1. ...if information about subpar performance is provided in the context of more assuring messages (feedback sandwich). | Cognitive Influences | 10 (16%) |
| 1. ...if multiple comparators provide consistent messaging. | Comparisons | 9 (15%) |
| 1. ...if it comes from an organization that is known to the recipient. | Trustworthiness/Credibility | 9 (15%) |
| 1. Feedback will be LESS effective if presented when no change in behaviour from the provider is suggested/required. | Goal Setting | 9 (15%) |
| 1. …when they introduce challenges to promote better learning. | Knowledge/Learning | 9 (15%) |
| 1. ...if it is internally generated and is also objective (e.g. self-conducted audit). | Nature of Data | 9 (15%) |
| 1. …if it is accompanied with educational training to allow for procedure to become automatized. | Single Hypotheses | 9 (15%) |
| 1. ...if important cues to behaviour are made salient. | Attract/Maintain Attention | 8 (13%) |
| 1. ...if during a protective (group) learning time. | Social Engagement | 8 (13%) |
| 1. …if they involve a self-persuasion component i.e. (self-generated reasons why the behaviour is worthwhile). | Single Hypotheses | 8 (13%) |
| 1. ...if it is presented in multiple sessions over time. | Feedback Timing | 8 (13%) |
| 1. ...when the comparator is clearly justified. | Goal Setting | 8 (13%) |
| 1. ...if both correct and incorrect instances of the behaviour are provided. | Motivation/Intention Issues | 8 (13%) |
| 1. ...if trend data is clear and in an undesired direction. | Nature of Data | 8 (13%) |
| 1. …when colour changes are purposeful and convey meaning. | Cognitive Load | 8 (13%) |
| 1. ...if benchmark comparisons are limited to the most important ones. | Comparisons | 8 (13%) |
| 1. ...if it incorporates data showing that population normative behaviour is trending in a direction consistent with the recommendations. | Comparisons | 8 (13%) |
| 1. ...if data about position/rank is provided, but not emphasized. | Comparisons | 7 (11%) |
| 1. ...if individuals persuade themselves that the message is credible. | Trustworthiness/Credibility | 7 (11%) |
| 1. ..when presented by someone (i.e. perhaps not the researcher) who enjoys an educational alliance with the participant. | Trustworthiness/Credibility | 7 (11%) |
| 1. …if they involve learning new behaviours in a group setting. | Social Engagement | 7 (11%) |
| 1. ..if it incorporates information from a barriers analysis conducted with low utilizers to determine the barriers to behaviour change. | Remove Barriers | 7 (11%) |
| 1. ...for high achievers when it involves comparison with the self. | Recipient Characteristics | 7 (11%) |
| 1. ...if information about opportunity costs is included. | Opportunity Costs | 7 (11%) |
| 1. …if practice feedback is used as a catalyst to encourage iterative , scenario-based feedback. | Knowledge/Learning | 7 (11%) |
| 1. ...if it includes more than simple knowledge about outcome probabilities. | Knowledge/Learning | 7 (11%) |
| 1. …if they include elements to enable patient requests of the desired behaviour (i.e., patient asks "did you wash your hands"?). | Memory | 7 (11%) |
| 1. ..if incorporates an emotional message underlining the desired behaviour | Memory | 7 (11%) |
| 1. ..if it is consistent with the explicit intentions of the target individual. | Motivation/Intention Issues | 6 (10%) |
| 1. Feedback interventions focusing on multiple behaviours will be more effective when behaviors are targeted for change sequentially before proceeding to the next behaviour. | About Aspects of Behaviour | 6 (10%) |
| 1. …if clinical procedure goals (e.g., reducing test ordering) are implemented first, before goals focused on overcall care (costs, overall morbidity). | Goal Setting | 6 (10%) |
| 1. …if they encourage learning of underlying concepts, rather than specific examples. | Knowledge/Learning | 6 (10%) |
| 1. ...if an aspirational goal is set. | Goal Setting | 6 (10%) |
| 1. ...if a writing component is part of a feedback reflection intervention. | Guide Reflection | 6 (10%) |
| 1. …if enablers and barriers are assessed after feedback is incorporated into practice. | Remove Barriers | 6 (10%) |
| 1. ...if target/ benchmark performance remains consistent over time. | Comparisons | 6 (10%) |
| 1. ...if it clearly and explicitly describes whether target feedback or comparators are closer to optimal performance (i.e. the "sign" of the feedback). | Enable Action Plans/Coping Strategies | 6 (10%) |
| 1. ...if it is not consistently negative. | Attack on Self-Identity | 5 (8%) |
| 1. when feedback specificity is presented at the optimal level (inverted U shape; is less effective if too specific or too general). | Feedback Specificity | 5 (8%) |
| 1. Effectiveness of feedback decreases according to the size of the provider group it summarizes increases. | Feedback Specificity | 5 (8%) |
| 1. …when it evokes specific, moment-to-moment safety goals, rather than encouraging a physician to engage in self-assessment after task completion. | Feedback Timing | 5 (8%) |
| 1. ...when presented at intervals that are long enough to prevent habituation. | Feedback Timing | 5 (8%) |
| 1. …when they encourage processing complementary to a person's typical strategy (for structured learners, focus on details. For detail learners, focus on structure). | Recipient Characteristics | 5 (8%) |
| 1. ...if as few graphs as possible are presented. | Cognitive Load | 5 (8%) |
| 1. …if the display is designed to minimize ink-to-information ratio. | Cognitive Load | 5 (8%) |
| 1. ...if fixed comparators, rather than those that change over time, are used. | Comparisons | 5 (8%) |
| 1. …if comparisons with norms are made so that numeric attributes become more highly evaluable. | Comparisons | 4 (7%) |
| 1. ...if it avoids being directive. | Enable Action Plans/Coping Strategies | 4 (7%) |
| 1. …if any social comparisons focus on specific individual patient cases rather than broad practice patterns | Feedback Specificity | 4 (7%) |
| 1. …if they include tests that encourage reflection on current knowledge | Guide Reflection | 4 (7%) |
| 1. …when behaviour rates are presented consistently | Nature of Data | 4 (7%) |
| 1. …when it incorporates standardized scenarios with controlled patient characteristics | Feedback Specificity | 3 (5%) |
| 1. …when its frequency is tied with end of practice administrative periods, rather than day-to-day practice | Feedback Timing | 3 (5%) |
| 1. Reminder messages will only be effective when knowledge is a barrier to behaviour | Memory | 3 (5%) |
| 1. …for those with a performance goal orientation if it does not involve comparison with others | Recipient Characteristics | 3 (5%) |
| 1. …if they target system components working at odds with each other | Remove Barriers | 3 (55) |
| 1. …when accompanied by information related to liability concerns | Remove Barriers | 3 (5%) |
| 1. …if it does not include absolute statements that could create liability issues | Remove Barriers | 3 (55) |
| 1. …if framed in terms of social conversations (memes) with which the recipient is familiar | Social Engagement | 3 (5%) |
| 1. …if group level data is provided only when the homogeneity of variance within the group is high | Comparisons | 3 (5%) |
| 1. …if frequent feedback is provided initially and made less frequent over time. | Feedback Timing | 2 (3%) |
| 1. …if incidence of type 1 errors (false positive or missing a test that should have been ordered) is low but incidence of type 2 errors (false negative or ordering a test that was not needed) is high. | About Aspects of Behaviour | 2 (3%) |
| 1. …for those with a mastery goal orientation if it involves comparison to others. | Recipient Characteristics | 2 (3%) |
| 1. feedback will be LESS effective when presented to those with greater expertise. | Recipient Characteristics | 2 (3%) |
| 1. People with higher organizational and job tenure are less likely to seek feedback. | Recipient Characteristics | 2 (3%) |
| 1. …if the recipient can respond to the feedback with "non-applicable” | User-Guided Experience | 2 (3%) |
| 1. …if it incorporates a gaming approach | Motivation/Intention Issues | 1 (2%) |
| 1. …for low self-esteem individuals, if negative feedback does not follow positive feedback | Recipient Characteristics | 1 (2%) |
| 1. …if the recipient generates a response immediately prior to receiving the feedback | Single Hypotheses | 1 (2%) |
| 1. …when guidance specifically addresses the sign of the feedback for that individual | Enable Action Plans/Coping Strategies | 1 (2%) |
| 1. …when not limited to correct/incorrect evaluations. | Knowledge/Learning | 0 (0%) |
| 1. …if it includes an unconditional incentive | Motivation/Intention Issues | 0 (0%) |
