# Supplementary material for: Informing the research agenda for optimizing audit and feedback interventions: results of a prioritization exercise
Source: BMC Med Res Methodol. 2021 Jan 13;21:20. doi: 10.1186/s12874-020-01195-5 (PMC7805176; doi:10.1186/s12874-020-01195-5)
Supplement: Supplementary file 2 — Additional file 2. Audit and Feedback Hypotheses Prioritization Exercise. [file 12874_2020_1195_MOESM2_ESM.docx]

**Appendix 2**

**Audit and Feedback Hypotheses Prioritization Exercise**

**Demographics**

1. In what country do you do most of your work?
2. I would describe my work as primarily:
   1. Research;
   2. Healthcare delivery
   3. Health policy;
   4. Health system administration;
   5. Other (Specify): _______________
3. What is your career level?
   1. New / Early (< 5 yrs)
   2. Mid-career (5-15 yrs)
   3. Senior (>15 yrs)
4. When you think about providing feedback in your area, does the feedback mostly involve…
   1. Feedback to individuals
   2. Feedback to units/practices
   3. Feedback to organizations
   4. Other (Specify): _______________
5. When you think about providing feedback in your area, are you primarily trying to effect…
   1. Change at an organizational level
   2. Change at a system level
   3. Other (Specify): _______________
6. In your work, what definition of feedback do you find most useful?

_______________________________________________

**Prioritization Exercise:**

**Instructions:**

As part of a larger initiative focused on understanding how different disciplines and theoretical backgrounds understand the challenges of providing feedback, we interviewed 28 different expert researchers about how they might improve typical health care feedback interventions and what theories guided their ideas. An extensive process of transcription, member-checking, and iterative development resulted in the 216 hypotheses included in the current prioritization task.

Each of these hypotheses is intended to represent an interesting, testable idea about how to improve the effectiveness of Audit and Feedback interventions.

We are asking you to select your **“top 50 hypotheses”** from the entire list.  We’d like you to make your choices based on the quality of the idea behind the hypothesis (as best you can interpret it), and its likelihood of advancing the field.  If an idea is poorly worded but interesting, consider selecting it (and perhaps leaving a comment about why you selected it).  Ideas that are unclear, uninteresting, or already well understood shouldn’t be selected no matter how clearly worded.  Please note that there is redundancy between some hypotheses. When this happens, please select your favorite (rather than all related hypotheses) and consider commenting on why you chose it over the others.

We anticipate that this exercise **will take approximately 60 minutes** to complete. We recommend you complete this exercise in one sitting; however, if you need to come back you may do so. We also recommend that you use a tablet or larger screen for easier viewing. You may wish to scan the themes before starting to make selections; navigation buttons should make it easy to get back to the start of the list.

| **Theme** | **Feedback will be more effective if…** | **Select with Checkbox; Optional comment** |
| --- | --- | --- |
| Cognitive Load | 1. ...if as few graphs as possible are presented. |  |
| Cognitive Load | 1. ...if graphical elements are without unnecessary depth elements. |  |
| Cognitive Load | 1. ...if text accompanying graphical components only describes information clearly related to the graphical content. |  |
| Cognitive Load | 1. ...if the graphical representations are clearly and consistently labelled. |  |
| Cognitive Load | 1. ...if the information explaining the A&F is clear and unambiguous. |  |
| Cognitive Load | 1. ...if educational messages are clearly presented. |  |
| Cognitive Load | 1. ...when the important comparisons are in proximity to one another. |  |
| Cognitive Load | 1. Feedback interventions involving multiple quality indicators will be more effective if the sign is consistent (i.e. higher numbers are better). |  |
| Cognitive Load | 1. ...if key messages are visually distinguished from supporting material. |  |
| Cognitive Load | 1. ...when different modes of information (e.g. graphics, text) are complementary, not redundant. |  |
| Cognitive Load | 1. ...if the interpretation to be drawn from the comparison to benchmark is made clear and explicit. |  |
| Cognitive Load | 1. ...if it provides a visually clear target rate. |  |
| Cognitive Load | 1. ...when colour changes are purposeful and convey meaning. |  |
| Cognitive Load | 1. ...when presenting absolute numbers as opposed to percentages. |  |
| Cognitive Load | 1. ...when designed to reduce cognitive load demands (e.g. include more white space, eliminate decimals, clear legend; left to right reading flow). |  |
| Cognitive Load | 1. Feedback interventions will be more effective when text is simplified and minimized. |  |
| Cognitive Load | 1. ...if the display is designed to minimize ink-to-information ratio. |  |
| Cognitive Load | 1. ...if only the most critical information is presented initially. |  |
| Cognitive Load | 1. ...if the focus is on only one specific behaviour at a time. |  |
| Cognitive Load | 1. ...when the reader is oriented to how to read the feedback. |  |
| Cognitive Load | 1. ...if it provides clear direction on the behaviour requiring change. |  |
| Comparison/Benchmark | 1. ...if a clear and explicit benchmark is provided. |  |
| Comparison/Benchmark | 1. ...if the benchmark comparison is accepted as a reasonable standard. |  |
| Comparison/Benchmark | 1. ...if the recipient agrees that the benchmark is relevant to them. |  |
| Comparison/Benchmark | 1. ...if it includes both local and overall norms. |  |
| Comparison/Benchmark | 1. ...if benchmark comparisons are limited to the most important ones. |  |
| Comparison/Benchmark | 1. ...if target/ benchmark performance remains consistent over time. |  |
| Comparison/General | 1. ...when a comparator is provided. |  |
| Comparison/General | 1. ...if the degree of difference between comparators is clear and made relevant. |  |
| Comparison/General | 1. ...if fixed comparators, rather than those that change over time, are used. |  |
| Comparison/General | 1. ...if multiple comparators provide consistent messaging. |  |
| Comparison/General | 1. ...if comparisons with norms are made so that numeric attributes become more highly evaluable. |  |
| Comparison/General | 1. Feedback interventions will be more effective if group level data is provided only when the homogeneity of variance within the group is high. |  |
| Comparison/General | 1. ...if it incorporates data showing that population normative behaviour is trending in a direction consistent with the recommendations. |  |
| Comparisons/Social | 1. ... when multiple individual physician practice data is presented along with the recipients’ data. |  |
| Comparisons/Social | 1. ... if any social comparisons are perceived as relevant and attainable. |  |
| Comparisons/Social | 1. ... if data about position/rank is provided, but not emphasized. |  |
| Comparisons/Self | 1. ... if it involves comparisons to the self. |  |
| Comparison/Specificity | 1. ... if the comparator is specific to the recipient’s own context/practice. |  |
| Providing Feedback Over Time | 1. ... if each episode of feedback includes multiple time points. |  |
| Providing Feedback Over Time | 1. ... if frequent feedback is provided initially and made less frequent over time. |  |
| Providing Feedback Over Time | 1. ... when it is presented continuously/as part of regular care. |  |
| Providing Feedback Over Time | 1. Feedback interventions will be more effective when they incorporate ways to track subsequent actions. |  |
| Providing Feedback Over Time | 1. Feedback interventions will be more effective if resulting patient outcomes over time support behaviour change. |  |
| Presenting Feedback Multiple Times | 1. ... if it is presented in multiple sessions over time. |  |
| Other Timing Issues | 1. ... when presented soon after the audited actions are taken. |  |
| Other Timing Issues | 1. ... when placed in the context of real-time comparison with peers. |  |
| Other Timing Issues | 1. ... if there is an immediate cue to action, during the patient encounter. |  |
| Other Timing Issues | 1. ... when it is available when the recipient is receptive to it (pull), rather than directed to them at a time not of their choosing (push). |  |
| Other Timing Issues | 1. ... when its frequency is tied with end of practice administrative periods, rather than day-to-day practice. |  |
| Other Timing Issues | 1. ... when it evokes specific, moment-to-moment safety goals, rather than encouraging a physician to engage in self-assessment after task completion. |  |
| Other Timing Issues | 1. ... if the frequency of the feedback is determined by the frequency of the target behaviour. |  |
| Other Timing Issues | 1. ... when presented at intervals that are long enough to prevent habituation. |  |
| Enable Action Plans/Coping Strategies | 1. ... if it suggests clear action plans. |  |
| Enable Action Plans/Coping Strategies | 1. ... if a response or action is required. |  |
| Enable Action Plans/Coping Strategies | 1. Feedback interventions will be more effective if they encourage people to use implementation intention strategies. |  |
| Enable Action Plans/Coping Strategies | 1. ... if it avoids being directive. |  |
| Enable Action Plans/Coping Strategies | 1. ... when guidance specifically addresses the sign of the FB for that individual. |  |
| Enable Action Plans/Coping Strategies | 1. ... if it clearly and explicitly describes whether target feedback or comparators are closer to optimal performance (i.e. the ‘sign’ of the feedback). |  |
| Social Engagement | 1. Feedback interventions will be more effective if they involve engaging recipients in social discussion about the feedback. |  |
| Social Engagement | 1. Feedback interventions will be more effective if they target communally determined behaviour change strategies (i.e. the group works together towards tipping points). |  |
| Social Engagement | 1. Feedback interventions will be more effective when they incorporate **facilitated** social discussions about the feedback. |  |
| Social Engagement | 1. Feedback interventions will be more effective if they encourage co-construction of goals among colleagues. |  |
| Social Engagement | 1. Feedback interventions will be more effective if they involve social group interaction within a safe/trusted environment. |  |
| Social Engagement | 1. Feedback interventions will be more effective if they address all relevant members of the practice team, not a single provider. |  |
| Social Engagement | 1. ...during a protective (group) learning time. |  |
| Social Engagement | 1. Feedback interventions will be more effective if they involve learning new behaviours in a group setting. |  |
| Social Engagement | 1. ... if disagreement with recommendations are explicitly acknowledged and addressed. |  |
| Social Engagement | 1. ... if framed in terms of social conversations (memes) with which the recipient is familiar. |  |
| Individual Provider Level | 1. ... if individual level provider data is provided. |  |
| Individual Provider Level | 1. Effectiveness of feedback decreases according to the size of the provider group it summarizes increases. |  |
| Individual Provider Level | 1. ... when it provides information on the appropriateness of individual decisions, not just frequency of behaviours. |  |
| Individual Provider Level | 1. ... if it is structured according to the most relevant data unit (e.g. individual, practice). |  |
| Individual Provider Level | 1. Feedback interventions will be more effective if individual level data is worded as a recommendation (e.g., in most cases, doing x is the best course of action) and aggregate level data is prescription (e.g., the guidelines states to do x). |  |
| Patient-Specific Feedback | 1. ... if patient-specific information is provided. |  |
| Patient-Specific Feedback | 1. ... if any social comparisons focus on specific individual patient cases rather than broad practice patterns. |  |
| Patient-Specific Feedback | 1. ... when it incorporates standardized scenarios with controlled patient characteristics. |  |
| Patient-Specific Feedback | 1. … when specific to patients most likely to benefit from the change in provider behaviour. |  |
| Ideal Level of Specificity | 1. …when feedback specificity is presented at the optimal level (inverted U shape; is less effective if too specific or too general). |  |
| Goal Setting | 1. ... if it is accompanied with a goal. |  |
| Goal Setting | 1. ... when target/goal/optimal rates are clear and explicit. |  |
| Goal Setting | 1. Feedback interventions will be more effective if the goal is above current performance. |  |
| Goal Setting | 1. ... if it clearly identifies a behaviour that should be improved. |  |
| Goal Setting | 1. Feedback will be LESS effective if presented when no change in behaviour from the provider is suggested/required. |  |
| Goal Setting | 1. ... if an aspirational goal is set. |  |
| Goal Setting | 1. ... when the comparator depicts the goal rather than a peer comparison. |  |
| Goal Setting | 1. Feedback based on groups will be more effective if individual members are personally committed to the group goal. |  |
| Goal Setting | 1. Feedback interventions will be more effective if clinical procedure goals (e.g., reducing test ordering) are implemented first, before goals focused on overcall care (costs, overall morbidity). |  |
| Goal Setting | 1. Feedback interventions will be more effective if the goals are believed to be reasonable and attainable. |  |
| Goal Setting | 1. ... when the comparator is clearly justified. |  |
| Goal Setting | 1. ... when it involves goals set/agreed to by the participant. |  |
| Goal Setting | 1. ... when it supports learner-determined rather than externally imposed goals. |  |
| Trustworthiness/Credibility | 1. ... if the feedback is provided by a trusted source. |  |
| Trustworthiness/Credibility | 1. …when presented by someone (i.e. perhaps not the researcher) who enjoys an educational alliance with the participant. |  |
| Trustworthiness/Credibility | 1. ... if it comes from an organization that is known to the recipient. |  |
| Trustworthiness/Credibility | 1. Feedback interventions will be more effective if the recipients of the feedback identify with the messenger of the feedback. |  |
| Trustworthiness/Credibility | 1. ... if a priori work is conducted to ensure acceptability of the benchmark and feedback. |  |
| Trustworthiness/Credibility | 1. ... if data come from sources similar to the recipient’s clinical practice. |  |
| Trustworthiness/Credibility | 1. ... if it is perceived to be without conflict of interest. |  |
| Trustworthiness/Credibility | 1. ... when recommendations related to the feedback are based on good quality evidence. |  |
| Trustworthiness/Credibility | 1. ... when origin of benchmarks is made clear. |  |
| Trustworthiness/Credibility | 1. ... if individuals persuade themselves that the message is credible. |  |
| Trustworthiness/Credibility | 1. Feedback interventions will be more effective if a record of success is established with “early win” goals prior to moving onto more challenging goals. |  |
| Motivation/Intention Issues | 1. ... when accompanied by incentive. |  |
| Motivation/Intention Issues | 1. ... if it includes an unconditional incentive. |  |
| Motivation/Intention Issues | 1. ... over time if it is accompanied with positive reinforcement to those who have improved their performance. |  |
| Motivation/Intention Issues | 1. ... if it can elicit a sense of achievement when a target is reached (achievement motivation). |  |
| Motivation/Intention Issues | 1. ... if it makes reference to performance successes in addition to providing clear direction on how to improve. |  |
| Motivation/Intention Issues | 1. ... if both correct and incorrect instances of the behaviour are provided. |  |
| Motivation/Intention Issues | 1. ... if they include motivational messages that are tailored to the individual provider. |  |
| Motivation/Intention Issues | 1. ... if it incorporates a gaming approach. |  |
| Motivation/Intention Issues | 1. Feedback interventions will be more effective if they make clear where the recipient is an outlier. |  |
| Motivation/Intention Issues | 1. Feedback interventions will be more effective if they target (triage) individuals who have motivation (intention) to change. |  |
| Motivation/Intention Issues | 1. ... if it is consistent with the explicit intentions of the target individual. |  |
| Knowledge/Learning | 1. ... when the practice gap is at least partly caused by a lack of knowledge. |  |
| Knowledge/Learning | 1. …if it creates opportunities to learn. |  |
| Knowledge/Learning | 1. ... if it is corrective (what was wrong, how to improve it). |  |
| Knowledge/Learning | 1. ... when not limited to correct/incorrect evaluations. |  |
| Knowledge/Learning | 1. Feedback interventions will be more effective when they introduce challenges to promote better learning. |  |
| Knowledge/Learning | 1. Feedback interventions will be more effective if they include active learning strategies (e.g. simulations, games with feedback). |  |
| Knowledge/Learning | 1. Feedback interventions will be more effective if they encourage learning of underlying concepts, rather than specific examples. |  |
| Knowledge/Learning | 1. Feedback interventions will be more effective if practice feedback is used as a catalyst to encourage iterative , scenario-based feedback. |  |
| Knowledge/Learning | 1. ... if it includes more than simple knowledge about outcome probabilities. |  |
| Remove Barriers | 1. ... if it addresses barriers and facilitations (drivers) to behaviour change. |  |
| Remove Barriers | 1. ... if it incorporates information from a barriers analysis conducted with low utilizers to determine the barriers to behaviour change. |  |
| Remove Barriers | 1. Feedback interventions will be more effective if they target system components working at odds with each other. |  |
| Remove Barriers | 1. Feedback interventions will be more effective if enablers and barriers are assessed after feedback is incorporated into practice. |  |
| Remove Barriers | 1. ... when accompanied by information related to liability concerns. |  |
| Remove Barriers | 1. ... if it does not include absolute statements that could create liability issues. |  |
| Remove Barriers | 1. ... if it incorporates messages specifically about barriers to the target behaviour. |  |
| Justify Need For Behaviour Change | 1. ... if accompanied by information about the importance of the behavior change. |  |
| Justify Need For Behaviour Change | 1. Feedback interventions will be more effective if the harms associated with incorrect behaviour in question are clearly indicated. |  |
| Justify Need For Behaviour Change | 1. ... when justified by improvements in patient care rather than cost savings. |  |
| Recipient Priorities | 1. ... if it is relevant to issues that are a priority for recipients. |  |
| Recipient Priorities | 1. ... when recipients believe that the target behaviour needs to change. |  |
| Recipient Priorities | 1. Feedback interventions will be more effective if they provide information sought by the recipient. |  |
| Recipient Priorities | 1. ... if targeted at a small number of the highest priority issues. |  |
| Recipient Characteristics | 1. …for high achievers when it involves comparison with the self. |  |
| Recipient Characteristics | 1. ... for those with a mastery goal orientation if it involves comparison to others. |  |
| Recipient Characteristics | 1. …for those with a performance goal orientation if it does not involve comparison with others. |  |
| Recipient Characteristics | 1. …for low self-esteem individuals, if negative feedback does not follow positive feedback. |  |
| Recipient Characteristics | 1. Feedback interventions will be more effective when they encourage processing complementary to a person’s typical strategy (for structured learners, focus on details. For detail learners, focus on structure). |  |
| Recipient Characteristics | 1. Feedback interventions will be more effective if they incorporate an understanding the communication style of the recipient. |  |
| Recipient Characteristics | 1. Feedback will be LESS effective when presented to those with greater expertise. |  |
| Recipient Characteristics | 1. People with higher organizational and job tenure are less likely to seek feedback. |  |
| Recipient Characteristics | 1. ... if targeted at those who are underperforming. |  |
| Cognitive Influences | 1. ... if emphasis is on what needs to be achieved (loss framing) as opposed to what was achieved (gain framing) (i.e., 20 % of your patients did not receive the proper prescription vs. 80% did receive the proper prescription). |  |
| Cognitive Influences | 1. ... when graphical representations of sub-par performance are displayed below, and good performance displayed above, a visual frame of reference |  |
| Cognitive Influences | 1. ... if noun descriptors rather than verbs are used in messaging (e.g., don’t be an over prescriber vs please prescribe less). |  |
| Cognitive Influences | 1. ... if information about subpar performance is provided in the context of more assuring messages (feedback sandwich). |  |
| Opportunity Costs | 1. ... when there are few costs to change behaviour. |  |
| Opportunity Costs | 1. ... if information about opportunity costs is included. |  |
| Opportunity Costs | 1. … if opportunity costs of engaging with the feedback are taken into account. |  |
| Attack On Self-Identity | 1. ... when measures are used to prevent a defensive response (e.g. providing other ‘reassuring’ messages as well, guiding self-reflection, etc.). |  |
| Attack On Self-Identity | 1. ... when it does not imply fault. |  |
| Attack On Self-Identity | 1. ... if it is non-punitive. |  |
| Attack On Self-Identity | 1. ... if it is not consistently negative. |  |
| Attack On Self-Identity | 1. ... if it elicits a clear affective response. |  |
| Attack On Self-Identity | 1. Feedback interventions will be more effective when they involve formative (identifying areas to help improve) rather than summative (performance only) assessment. |  |
| About Aspects of Behaviour | 1. Feedback about behaviour will be more effective for behaviors that are easy compared to those that are harder to do. |  |
| About Aspects of Behaviour | 1. Feedback interventions involving stopping behaviours will be more effective if they involve persuasive components. |  |
| About Aspects of Behaviour | 1. Feedback interventions involving starting new behaviours will be more effective if they involve reminders/prompts. |  |
| About Aspects of Behaviour | 1. ... if it is about a behaviour that does not rely on others. |  |
| About Aspects of Behaviour | 1. ... when it addresses a behaviour that is relevant to the current patient. |  |
| About Aspects of Behaviour | 1. ... if incidence of type 1 errors (false positive or missing a test that should have been ordered) is low but incidence of type 2 errors (false negative or ordering a test that was not needed) is high. |  |
| About Aspects of Behaviour | 1. Feedback interventions focusing on multiple behaviours will be more effective when behaviors are targeted for change sequentially before proceeding to the next behaviour. |  |
| Reminders | 1. Feedback interventions will be more effective if they also incorporate reminders. |  |
| Reminders | 1. ... if the reminder messages are presented in real time/point of care. |  |
| Reminders | 1. Feedback interventions will be more effective if they include elements to enable patient requests of the desired behaviour (i.e., patient asks "did you wash your hands"?). |  |
| Reminders | 1. Reminder messages will only be effective when knowledge is a barrier to behaviour. |  |
| Memory/General | 1. ... if incorporates an emotional message underlining the desired behaviour. |  |
| Memory/General | 1. Feedback interventions will be more effective if they include memorable/salient messages. |  |
| Attract/Maintain Attention | 1. ... when it is sufficiently salient and receives sufficient attention. |  |
| Attract/Maintain Attention | 1. Feedback interventions will be more effective if they encourage engagement with the data. |  |
| Attract/Maintain Attention | 1. ... when presented in a clear and aesthetically pleasing way. |  |
| Attract/Maintain Attention | 1. ... if important cues to behaviour are made salient. |  |
| Attract/Maintain Attention | 1. ... if the recipient reads / processes it. |  |
| Guide Reflection | 1. ... if it involves a personal reflection component. |  |
| Guide Reflection | 1. ... if a writing component is part of a feedback reflection intervention. |  |
| Guide Reflection | 1. ... if reflection occurs soon after feedback. |  |
| Guide Reflection | 1. ... if it encourages reflection on the original pattern of behaviour. |  |
| Guide Reflection | 1. Feedback interventions will be more effective if they include tests that encourage reflection on current knowledge. |  |
| Guide Reflection | 1. Feedback interventions will be more effective when recipients internalize and act on the feedback, rather than only responding to a system prompt. |  |
| User-Guided Experience | 1. ... if it includes multi-layered feedback which begins with high-level feedback and then drills down to the details. |  |
| User-Guided Experience | 1. ...if the recipient can respond to the feedback with "non-applicable". |  |
| User-Guided Experience | 1. ... when it can be customized by the recipient. |  |
| User-Guided Experience | 1. ... if more detailed information is available on demand. |  |
| Nature of Data | 1. ... if trend data is clear and in an undesired direction. |  |
| Nature of Data | 1. ... if trend data are sufficiently stable to facilitate interpretation. |  |
| Nature of Data | 1. ... if graphical representation displays the variability of data in order to indicate the error or uncertainty (i.e., confidence intervals). |  |
| Nature of Data | 1. ... if it is internally generated and is also objective (e.g. self-conducted audit). |  |
| Nature of Data | 1. ... when behaviour rates are presented consistently. |  |
| Self-Efficacy/Control | 1. ... if the behaviour is under the control of the recipient. |  |
| Self-Efficacy/Control | 1. ... if it is expected that recipient behaviour change will result in improvements. |  |
| Self-Efficacy/Control | 1. Feedback interventions will be more effective if the recipients have the capabilities to respond to the feedback. |  |
| Self-Efficacy/Control | 1. ... it facilitates respect, feelings of control over the learning agenda. |  |
| Self-Efficacy/Control | 1. ... when recipients believe the change is THEIR idea. |  |
| Environment | 1. ... if it incorporates the typical clinical encounter decisions in the specific context. |  |
| Environment | 1. ... if incorporated into familiar processes of care. |  |
| Environment | 1. Feedback interventions will be more effective if the environment encourages the desired behaviour as the default. |  |
| Decision Processes or Conceptual Model | 1. ... if designed with a clear understanding of the decision making process underlying the behaviour to be changed. |  |
| Decision Processes or Conceptual Model | 1. Feedback interventions will be more effective if recipients are involved in the design/development of the feedback intervention. |  |
| Decision Processes or Conceptual Model | 1. Feedback interventions will be more effective if targets are made aware of the involvement of other stakeholder groups in the development process. |  |
| In-Person Feedback | 1. ... when it is in person (can be presented in a manner that is responsive to the situation). |  |
| Responding to Feedback Providers | 1. Feedback interventions will be more effective if they allow an opportunity to indicate why a recommended action wasn’t taken. |  |
| Responding to Feedback Providers | 1. Feedback interventions will be more effective if they allow the recipient to respond to the feedback providers. |  |
| Single Hypotheses | 1. Feedback interventions will be more effective if they imply some kind of extended commitment (e.g. agreeing to a future communication, follow-up). |  |
| Single Hypotheses | 1. ... if the recipient generates a response immediately prior to receiving the feedback. |  |
| Single Hypotheses | 1. ... if it includes stratification by common 'alibi' variables (i.e., demonstrating that "my patients are not sicker). |  |
| Single Hypotheses | 1. ... if it is accompanied with educational training to allow for procedure to become automatized. |  |
| Single Hypotheses | 1. Feedback interventions will be more effective if they involve demonstrations of the behaviour. |  |
| Single Hypotheses | 1. Feedback interventions will be more effective if the goal is made public. |  |
| Single Hypotheses | 1. Feedback interventions will be more effective if they involve a self-persuasion component i.e. (self-generated reasons why the behaviour is worthwhile). |  |
| Single Hypotheses | 1. ... if it focuses on patient outcome measures rather than process measures. |  |
| Single Hypotheses | 1. ... if it is provided to the intended target for behavior change. |  |
| Single Hypotheses | 1. ... if it contains multi-modal presentation (both text and graphs.) |  |
